# Barriers and facilitators to the implementation of person‐centred care in different healthcare contexts

**DOI:** 10.1111/scs.12376

**Published:** 2016-11-08

**Authors:** Lucy Moore, Nicky Britten, Doris Lydahl, Öncel Naldemirci, Mark Elam, Axel Wolf

**Affiliations:** ^1^ Institute of Health Research University of Exeter Medical School Exeter UK; ^2^ Department of Sociology and Work Sciences University of Gothenburg Gothenburg Sweden; ^3^ Institute of Health Care Sciences Sahlgrenska Academy University of Gothenburg Gothenburg Sweden; ^4^ Gothenburg Centre for Person Centred Care (GPCC) University of Gothenburg Gothenburg Sweden

**Keywords:** facilitators, intervention research, barriers, long‐term conditions, nurse–patient relationships, nurse–physician relationships, person‐centred care, qualitative methods

## Abstract

**Background:**

To empower patients and improve the quality of care, policy‐makers increasingly adopt systems to enhance person‐centred care. Although models of person‐centredness and patient‐centredness vary, respecting the needs and preferences of individuals receiving care is paramount. In Sweden, as in other countries, healthcare providers seek to improve person‐centred principles and address gaps in practice. Consequently, researchers at the University of Gothenburg Centre for Person‐Centred Care are currently delivering person‐centred interventions employing a framework that incorporates three routines. These include eliciting the patient's narrative, agreeing a partnership with shared goals between patient and professional, and safeguarding this through documentation.

**Aim:**

To explore the barriers and facilitators to the delivery of person‐centred care interventions, in different contexts.

**Method:**

Qualitative interviews were conducted with a purposeful sample of 18 researchers from seven research studies across contrasting healthcare settings. Interviews were transcribed, translated and thematically analysed, adopting some basic features of grounded theory.

**Ethical issues:**

The ethical code of conduct was followed and conformed to the ethical guidelines adopted by the Swedish Research Council.

**Results:**

Barriers to the implementation of person‐centred care covered three themes: traditional practices and structures; sceptical, stereotypical attitudes from professionals; and factors related to the development of person‐centred interventions. Facilitators included organisational factors, leadership and training and an enabling attitude and approach by professionals. Trained project managers, patients taking an active role in research and adaptive strategies by researchers all helped person‐centred care delivery.

**Conclusion:**

At the University of Gothenburg, a model of person‐centred care is being initiated and integrated into practice through research. Knowledgeable, well‐trained professionals facilitate the routines of narrative elicitation and partnership. Strong leadership and adaptive strategies are important for overcoming existing practices, routines and methods of documentation. This study provides guidance for practitioners when delivering and adapting person‐centred care in different contexts.

## Introduction

Patient‐centred care is professed to have more evangelists than practitioners 1, yet models of person‐centredness and patient‐centredness have become increasingly adopted by policy‐makers 2, 3. Recent debates about patient‐centredness and person‐centredness demonstrate a shift towards inclusivity and equity in the professional–patient relationship 4. In keeping with other healthcare providers internationally, Swedish healthcare professionals seek to strengthen the position of patients and their participation in care 5.

Although patient‐centred care and person‐centred care (PCC) are frequently conflated in the literature, in both, professionals are encouraged to acknowledge the patient as an equal partner in the development and assessment of their care 6, 7. Evidence suggests that PCC can be delivered effectively 8, and patients with long‐term conditions benefit from this approach 9, 10, 11. Moreover, researchers report that PCC can be facilitated through effective leadership 12, 13 and by knowledgeable professionals with sound communication skills 7.

The uptake of PCC remains sporadic as barriers are identified as well as facilitators 14, 15, 16, 17, 18. As McCormack 19 suggests the context of the care environment has the greatest potential to restrict or support PCC in practice. The implementation of PCC can also vary depending upon patient populations, providers of care and settings and how professionals and patients understand what constitutes caring 20. Additional challenges to PCC include professional practice, beliefs and cultures 21; professionals erroneously believing that they are practicing PCC; or reverting to disease‐centred care when under pressure 15. How PCC is translated into practice remains challenging 22 and person‐centredness needs to be considered in the wider context in terms of the care environment and beyond 17.

The implementation of PCC poses challenges. Few studies describe how healthcare professionals can become proficient, well‐trained PCC practitioners and integrate theory into practice in contrasting settings 23. Less is known about what we can learn from implementing and evaluating PCC in the context of routine care, patients’ priorities and personal needs. Addressing these gaps is important to gain insight into the factors that help or hinder understanding, acceptance and implementation.

To improve the practice of PCC, researchers at the University of Gothenburg, Sweden, successfully obtained national funding to establish the Centre for Person‐Centred Care (GPCC). At GPCC, about 40 research projects are currently investigating PCC from the perspective of people with long‐term conditions and health professionals delivering care 24. These studies are based in various hospital, primary care and community settings in Sweden. At GPCC, the term person‐centred care (PCC), as opposed to patient‐centred care is preferred, as this acknowledges the person behind the patient 12. This centre funds and conducts research in the field of PCC, in a variety of healthcare settings, anchored within a model of PCC that has clear philosophical and practical guidance. The GPCC model of PCC relies upon three simple routines 15, 25. The first routine elicits the patient narrative or subjective account of the person's illness experience, strengths and future plans. The second agrees a partnership with shared decisions and goals between professional, patient and often their relatives, and the third routine ensures this partnership and narrative is documented. 12. These routines were initially tested in a controlled clinical study of people hospitalised for worsening chronic heart failure and further developed in a recent randomised clinical study on acute coronary syndrome (ACS) 26, referred to here as the index project. This subsequently formed the basis for the GPCC model.

## The study

### Aim

The aim of this study was to explore the barriers and facilitators to the delivery of PCC interventions, as defined by the GPCC model, in a range of contrasting healthcare settings.

### Methods

The research was a qualitative study of GPCC researchers involved in implementing the GPCC model. It was designed and conducted by an international team based in Gothenburg, Sweden and Exeter, UK. Of approximately 40 projects at GPCC, seven were purposefully selected because they represented the broad scope of healthcare provision within primary, secondary and tertiary services and provided a kaleidoscope view of actual and potential barriers and facilitators in different stages of implementation. These projects were as follows: acute coronary syndrome (ACS; the index project), irritable bowel syndrome (IBS), neurogenic communication disorders, healthy ageing in migrant communities, patient participation in hypertension treatment, psychosis and osteopathic fractures. Researchers’ descriptions of barriers and facilitators for PCC and the routines of narrative, partnership and documentation are described across projects. In this study, the term barrier refers to the challenges considered by researchers and how they tried to overcome these challenges when implementing PCC in different contexts.

### Interviews

Three researchers in Gothenburg (ME, DL & ON) conducted interviews with 18 researchers, whose first language was not English, in 2013 and 2014. There were two or three interviewees from each of the seven projects. Interviews conducted in Swedish were translated into English while those conducted in English were transcribed. Interviewees were contacted by email and interviewed in their office or the Department of Sociology and Work Science, University of Gothenburg, where informed consent was given. The duration of interviews was 45–78 minutes.

A semistructured interview guide was developed by the research team to generate extended and reflective answers on the definition of PCC and how PCC was operationalised. This guide incorporated questions such as: How would you explain PCC to someone who had never heard of it? and Tell me about a particular example of effective PCC in your project? This was followed by probing questions such as: What does PCC look like in your project in terms of staff involved, patients’ experiences, ways of working? There were specific questions about barriers and facilitators: What do you think would assist or facilitate person‐centred care? Do you have examples of situations where difficulties arose? Interviewees describe their professional experience of PCC in general as well as their experience of implementing PCC in their GPCC project.

### Analysis

Interviews were thematically analysed, adopting some basic features of grounded theory, employing an interpretive approach to prioritise participants’ accounts, perspectives and experiences of PCC 27. This included open coding, constant comparison, looking for similarities, differences and patterns within the data and developing theoretical insights. Through an iterative process, two UK‐based researchers (LM and NB) drew up a coding frame of interviewees’ accounts of the meaning and implementation of PCC in their research and practice. This initially incorporated six codes: descriptions of the patient's account, professional philosophy, thinking about differences, the approach to working for PCC, thinking about difficulties, the group and population and the intervention. Early analysis of the interviews in 2013 guided the subsequent selection of projects in 2014 and future data collection. Vignettes, using examples of interviewees’ descriptions of PCC in practice and challenges encountered, were used to aid discussions, identify deviant cases and reach a consensus. The analysis was a collaborative process, shared and developed with the rest of the team. At a team meeting in Gothenburg, a comparative framework for barriers and facilitators was created and formed the basis for three tables. These tables were presented to a representative group of interviewees from all projects to review. A final draft of the paper was sent to the principal investigators for checking, prior to submission.

### Ethical considerations

The ethical code of conduct was followed and conformed to the ethical guidelines adopted by the Swedish Research Council. In accordance with Swedish ethical protocol, no ethical approval was required. However, all participants gave their informed consent and were assigned a number to ensure anonymity.

### Findings

The findings describe the barriers and facilitators to PCC reported by researchers at GPCC and are summarised in three tables (Table 1).

**Table 1:** Characteristics of selected GPCC projects

Category	Acute coronary syndrome (index project)	Irritable bowel syndrome	Psychosis	Osteopathic fractures	Patient participation in hypertension treatment	Neurogenic communication disorders	Healthy ageing in migrant communities
Intervention population	People with acute CAD Symptomatic – to show effect	Men and women with IBS and no biological markers	People with psychosis	Older people with osteofracture and pain Average age 84	People over 30 years medically treated for hypertension	People with neurological disease and HCP in nursing homes	Foreign – born older persons
Setting and speciality	Acute care and primary care Cardiology	Acute care and primary care Medicine	Four acute in‐care units Psychiatry	Acute care, community and person's home Orthopaedics	Medical outpatient clinic and primary care Medicine	Nursing homes Speech Language therapy	Community centre and person's home Occupational science and health promotion In the context of migration
Intervention purpose and outcome	To increase self‐efficacy and resumption of activities	Identify gender differences Understand illness perspectives Develop PCC dietary advice	Understand person's perspective and create a plan for social resources Reduce symptom burden, involuntary injections hospital stay and overall burden on ward	Reduce pain and restore function/activity through support, rehabilitation and activity prescription Reduce length of stay in acute care – cost efficiency	To design, develop and evaluate an interactive mobile phone‐based system to support self‐management of hypertension	The communicative competence of HCP as a resource in PCC for people with communication disorders Emphasise need for PCC trained HCP	Promote health and normal ageing Empower participants and lift strengths through peer support Emphasise contextual perspectives
Intervention status	Completed	Planning an intervention	Planning an intervention	Ongoing	Completed	Ongoing	Completed and under evaluation
Design of research	RCT	Qualitative (group interviews and questionnaire) RCT planned	Before and after study (pre measurement of ward culture, patient satisfaction and empowerment (focus groups and questionnaires)	RCT	Focus groups validation study. Before and after study of self‐reports and video recordings of consultations	Mixed method design (questionnaires and video recordings before, during and after the intervention with HCP used to evaluate effect of training	RCT and implementation research

CAD, coronary artery disease; IBS, irritable bowel syndrome; PCC, person‐centred care; RCT, randomised control trial; HCP, healthcare professional.

### Characteristics of the seven projects

The projects were chosen to provide a range of settings, populations and goals (Table 1). The projects were in various stages of the intervention process and delivered by researchers from a wide range of professional backgrounds. These interventions targeted a diverse group of people with acute or chronic conditions, as well as preventative strategies for older people from migrant communities. Interventions were based within a single setting or transferred from acute care through to primary and community care. The research rationale, design and outcome measures necessarily varied depending upon certain contextual factors and individual needs of patients and professionals. PCC interventions focused on individual patients, groups or dyads with an educational and training component for professionals, the intervention population or both. In the following section, barriers and facilitators to the implementation of PCC, identified by researchers, are described in detail and summarised in Tables 2 and 3.

**Table 2:** Summary of barriers for PCC

Projects	Traditional practices and structures	Time constraints	Professional attitudes	Population characteristics	Design and documentation
Acute coronary syndrome (index project)	Objective measures – technology and screens Professional goal not persons Removed hospital walls – not private	On ward rushing and falling back into usual care	Claim PCC when not Sceptical and negative	Need reasonably symptomatic patients Obliged to find interested staff	Challenge to establish care chain from hospital to primary care Documentation challenging for new staff – writing care plan and new communication approach to make dialogue possible
Irritable bowel Syndrome	Objective measures – positivistic culture Patients and staff – regulated and programmed	Fast pace – difficult to prioritise PCC	Patients seen as malingerers – creates mistrust	Vulnerable group – difficult to treat– subjective symptoms	Group intervention not individual – debatable whether PCC Documentation – fragmented
Psychosis	Treatment centred on medicine – symptom control device in involuntary care Locked doors on wards – no privacy	Time needed to let medication take effect	Patients not seen as ‘person’ – do not share reality with professional	Illness and symptoms – decrease autonomy, cognition and insight – dangerous goals puts restraint on professionals	Implementation of research and ward environment collide Mixed method PCC research complex Documentation – lacking
Osteopathic fractures	Physician led – standardised prescribing and lack of cooperation Professional goal not patients	Workload and high turnover in surgery PCC increases patient workload	Nurses do not listen or do not hear narrative Nurses think they have no power	Patients admitted acutely ill and sedated – often frail and undemanding	Documentation – problematic
Patient participation in hypertension treatment	Professional agenda set over decades	Time needed for patients to understand importance of following treatment	Professional agenda needs to change to support new conversation with patient and continuity of care	Patients’ difficulty seeing relationship between symptoms and signs	Documentation in the form of database outputs and graphs.
Neurogenic communication disorders	Staff culture – focussing on speech not on alternative communication aids	Nursing home workload Time needed to talk to a person who cannot speak	Claim PCC when not Disinterested or lack knowledge	Vulnerability of residents (aphasic, frail – end of life)	Hard work for HCP being filmed High turnover of HCP – cannot complete filming with all dyads Documentation – difficult
Healthy ageing in migrant communities	Medical positivist culture – give knowledge rather than listen Professional goal not persons	Time needed to reach person's goal	Claim PCC when not Stereotyping – ageing people or talking about people not with them	Language skills –translation and interpreting problems – Mistrust of officials	Measuring PCC challenging

BP, blood pressure; GPCC, University of Gothenburg centre for person‐centred care; PCC, person‐centred care; HCP, healthcare professional.

**Table 3:** Summary of facilitators for PCC

Projects	Organisation and leadership	PCC training and education	Professional attitude and approach	Delivery of research
Acute coronary syndrome (index project)	Leadership emphasises and values PCC Index project receives attention as pioneer	Training in PCC communication	Interested and positive Seeing patient as person – listening to narrative	Primary care – tradition to have a dialogue Well‐trained managers in primary care – patient's care plan agreed and continued into primary care
Irritable bowel syndrome	Leadership – act as forerunner for PCC Multidisciplinary team work and power shift – professionals as equals	Training in PCC communication and philosophical underpinnings	Seeing patient as person – equal partner	Participatory design – patients share symptom graph
Psychosis	Bottom up and top down recognition for change – good information channels Multidisciplinary team work – cooperating with people who are successful for PCC	Training in communication by psychologist	Seeing person as capable – equal partner Willing to change communication style – mindset for PCC Able to work with difficult symptoms – create trusting relationship	Participatory design – agree social resource group with relatives for when symptoms abate
Osteopathic fractures	GPCC makes PCC explicit – increase knowledge through research studies Multidisciplinary team working – staff work ‘with physician not under’	Maintain and develop PCC through education and research Training to find out patient's motivation and resources	Interested and involved – believe in skills and PCC Listening to patient in different way – ‘hearing’	Positive effect of PCC intervention – visible with older population Participatory design – intention to involve patients along the way – perception of intervention and PCC
Patient participation in hypertension treatment	Leading from top of the organisation down for PCC – communicating how you look at human being in the context of care	Patients self‐reports used as a base for consultations – professionals become advisor for discussion and conversation	Patient seen as person – equal partner and take initiative back – coproduce	Project connected to primary care – patients have a system to be connected with in everyday life Participatory design – interdisciplinary group and patients create tool – mobile phone system supports patients involvement in consultations – BP significantly decreased
Neurogenic communication disorders	Multidisciplinary team meetings for PCC	PCC education – makes staff knowledgeable – supports learning Training in communication for staff in dyad with resident	Interested in PCC – staff have personality for it. Seeing person as communication partner – a learning process Seeing person as active agent in care	Participatory design – staff and person with aphasia affects the intervention and data collection
Healthy ageing in migrant communities	Leadership for PCC – seeing coworkers as people Multidisciplinary team working	Training for group leader – helps group grow	Focus on person's goal not professional expertise	Effective project managers Researchers translate material and documents to mediate the research and simultaneous interpreter improves group dynamic in the intervention

BP, blood pressure; GPCC, the University of Gothenburg centre for person‐centred care; PCC, person‐centred care.

### Barriers and facilitators of PCC

#### Barriers

Across all projects, three themes emerged from the analysis related to (1) traditional practices and structures, (2) attitudes and (3) development of the PCC intervention. Barriers are described in relation to the GPCC model.

#### Traditional practices and structures

Several researchers reported working within a positivist healthcare tradition, described by one interviewee as the ‘heavy machinery of their healthcare service’

it's built up around the biomedical paradigm. So it's very difficult to, er, change this, but I guess just focusing on the patient narrative is kind of a power shift, ‘cause then you um, you, just giving the patients the opportunity to speak and give the space for them to share experiences (R6).

Professionals working according to traditional care pathways restrict the freedom to do things differently than ‘usual care’. The ability to work flexibly and use different strategies was described by some researchers as a prerequisite for PCC. Existing power relationships, invariably built around physicians, were perceived as troublesome to change. The surgical setting proved a particularly tough climate for PCC with ‘a high patient turnover’ and standardised prescribing (R8).

Researchers described situations where patients were programmed to speak and act in a particular way that restricted the freedom to engage in a person‐centred dialogue:

Some patients you want them to speak freely and to take up things that they feel are of importance, but they are so programmed, I mean they've been to the doctor and the nurse before, they know what they are supposed to say in this context, and what kind of things they are supposed to bring up (R6)

Some professionals prioritised ‘objective’ and ‘medicalised’ aspects of IBS symptoms. Patients then became passive recipients of professional agendas, leaving the patient narrative lost. For example, professionals in health promotion ‘give out knowledge rather than listen’ to migrant groups (R4) or focused only on speech for communicating rather than alternative nonverbal communication aids to help elicit the narrative for people with neurogenic communication disorders. Several researchers described the paradox of advances in care that proved beneficial as well as a barrier to PCC. Biomedical knowledge and technological aspects of care, although a necessity for some professionals, posed challenges for the PCC approach. In some cases, professionals prioritised ‘objective data’ and screens in cardiology (R12) or medications in involuntary care to control psychotic symptoms, rather than immediately listen to the patient's narrative.

a person arrives to the clinic with police transport and is placed in involuntary care, the first thing to do is try to get a grip of the symptoms, and it's not until that is underway that it's actually going to be possible to have this interview with the patient to create the care plan (R14)

For this researcher, only once the Medication is administered are they able to talk, ‘share worlds’ and elicit the narrative.

Some researchers also described professionals with preset goals who affected the routine of partnership. Often a balance had to be struck between professionals and patients taking control and professionals collaborating together in partnership:

if the goal is unrealistic. We obviously…if someone says ‘When I had this, I wanna have had this…’ I will…. I will reduce weight by 50%, I will do this and in 2 months I will do…’ And then you can say that ‘Well, what you want to do sounds fine but it's not possible’. We have to break it down to other steps and ‘why not this or that (R7)

One researcher described the importance of getting ‘a new context in the conversation’, between professionals and people with hypertension. In this study, patients needed to take the initiative and share their data as co‐creators. Overcoming barriers to PCC required nurses, as well as patients, to adopt a different role.

we have our individual, er, tasks to perform, to do this, to, to investigate the patient from the patient perspective, to investigate and, and measure the context of the patient, and to find that treatment goal, and all that, and of course we should still do what the doctor tells us, I mean …But we have a different role and we need to work together with the physician, not *under* the physician. Which is what usually happens (R8).

Two researchers also identified physical structures that proved an obstacle to elucidating a narrative and developing a partnership. Open plan areas lacked personal spaces for listening to sensitive issues in hospitals, where a lack of private rooms affected communication between professionals and patients (R7). Conversely, in psychiatry, the walls placed around the person for their safety and safety of others needed to be aligned with patients’ wishes. The structure of the ward was described as ‘not person‐centred architecture at all’ (R14).

### Time constraints

For several researchers, insufficient time constrained PCC delivery. It took time to participate in training, education and develop partnerships for professionals and patients. Similarly, professionals needed to learn new communication techniques for patients with communication disorders or work with older migrants, through translators. The fast pace of healthcare activities, in some contexts, made PCC implementation difficult. However, once embedded, several researchers described how PCC saved time when patients took responsibility for their own care or when a person's goals were effectively elucidated and supported.

### Professional attitudes

Professionals’ attitudes were a barrier to PCC, whereby consciously or unconsciously, they slipped back into ‘usual care’ or lacked the interest, knowledge or commitment. Several researchers described working with professionals who said they were practicing PCC when they were not. Maintaining a PCC approach required conscious effort. The routine of partnership was affected when professionals ‘do not see the person’ and argue with a patient with psychosis, rather than calmly listening to the narrative. Another researcher described professionals viewing patients with IBS as ‘malingerers’ who engendered mistrust (R6). Personal politics towards both co‐workers and patients affected PCC delivery:

there's an attitude lacking. And I think it's hard, I think it's *really* hard to, to work with attitudes, um, but I think that's what we're doing. Because I told you that, um, the people that we have been training with, that have been in our previous studies …we think that these are the persons, the persons that are really interested in communication and wants to learn. And if you work with a person that thinks he or she knows everything already, um, ‘No, I'm doing this and I'm doing that’, then that will be a totally different thing. More of a challenge (R17)

Attempting to change attitudes that were sceptical and stereotypical was difficult and required continuous attention and engagement with PCC routines. Professionals needed to be repeatedly reminded that the patient is a person because it was easy to fall back into old ways of thinking (R7).

### Population characteristics, design and documentation

Researchers identified barriers specific to the development of PCC interventions including recruitment, study population, research design and setting. This included having to recruit reasonably symptomatic patients and feeling an obligation to find interested professionals. A high turnover of professionals and workload in nursing homes for instance restricted professionals’ participation in lengthy video recordings with residents (R3). As another researcher stated

the clinic is, um, working towards getting more research in general in different projects like, er, involving this, you know, this patient group. And that's a problem because there's always something going on in the wards that is maybe sometimes colliding with what we would want with the intervention (R14)

Researchers were working with a population with diverse health problems and needs. For example, patients with psychosis experienced decreased autonomy and insight, with goals that might be dangerous or unrealistic (R15). A vulnerable study population also proved a barrier to PCC where patients were aphasic and/or frail, acutely ill, sedated and undemanding. Similarly, the partnership and narrative components of PCC were demanding when working with people who mistrusted officials based on previous encounters and language barriers.

PCC interventions were diverse with researchers working with individuals, groups and dyads. Researchers considered whether a group‐centred intervention was less person‐centred than an intervention delivered to individuals. Others described balancing stereotypical images of ageing people born abroad and acknowledging the similarities within the group as well as differences.

Structuring and documenting the narrative and partnership, as a core component of PCC, was described as difficult by researchers across some projects. Although this documentation was important to demonstrate that patients had received PCC, it was described as fragmented, poorly developed and lacking. Professionals wrote in separate areas and in contrasting ways when working with existing documentation. As one researcher suggested:

it's part of the intervention in the departments – is a sort of communication plan for the homes, which needs to be available to all staff and needs to be documented. On the one hand, it's about good strategies – but also more personal ones. There's a person who's spent a lot of time in the forests and fields and they like talking about it. So, that type of thing, in other words. Where perhaps we could squeeze it in some way (R3)

In summary, the barriers to PCC involved a biomedical approach that was deemed strong and well developed and traditional professional attitudes were considered difficult to change. Researchers described barriers related to the intervention design, population, care setting and existing documentation systems.

### Facilitators

Despite the barriers outlined above, researchers also identified where organisational factors, leadership and training, the attitude and approach of professionals and how PCC was delivered across projects helped rather than hindered the delivery of PCC (See Table 3).

### Organisation and leadership

For several researchers, working with GPCC provided leadership where projects were stimulated by the emphasis on PCC research. This strengthened and highlighted PCC, with the index project serving as an example of how the PCC routines could be implemented in practice. However, GPCC definitions of PCC were necessarily modified and adapted in different care contexts. The majority of researchers described leadership styles that instigated organisational change, emphasising PCC values and working practice, and interprofessional team working. Leaders served as role models and forerunners, consistently working in a PCC way. For one researcher, personal politics played a part:

How you view your, er, co‐workers, I believe in a person‐centred leadership as well, that every co‐worker needs to be regarded as a person and be seen as who they are and what they want to do (R16)

Multidisciplinary teamwork meant collaborating with patients, health professionals and experts, across organisations. This required a horizontal balance of power to implement PCC successfully. Certain environments lent themselves to PCC implementation such as primary care where researchers described a system for patients to connect to in their ‘everyday life’ (R9) or where it was natural to carry out a dialogue with patients (R12). For the index project, a care plan guaranteed the care chain and ensured patients had a ‘new partner’ in primary care (R5).

### PCC training and education

Professional training and education, for the successful implementation of PCC, was commonly cited across projects. Success was dependent upon well‐trained professionals with a genuine knowledge of the patient and how to practice communicating in a PCC way. Practitioners learnt to communicate effectively in partnership with people with a neurogenic disorder or learnt to become an ‘advisor’ for people with hypertension. As one researcher stated, professionals attended training courses to learn how to work effectively and lead a discussion with older migrants to promote peer support:

The group leader, I'd like to state that we have the role of the group leader and how to make the group grow, not down, yes we have a training‐course in that and that's what's important. yes, I'd probably say that.

I: So the strategies are important?

Yes, they are, for it to be person‐centred at all (R10)

Trained professionals involved the resident and family member, to help obtain the person's narrative or goal. Practitioners fostered partnerships using different strategies and tools for different patients related to culture, communication, linguistic ability or personal goals.

### Professional attitudes and approach

Researchers cited the importance of interested staff and a committed group of professionals with a positive attitude that allowed for trust and partnership building. Successful implementation was dependent upon professionals’ skilfulness shown in listening ‘in a different way’ to patients (R8), confirming, documenting and repeatedly acknowledging the person's narrative. Seeing the patient as a person who is active and a capable or equal partner was cited as a common facilitator. For one researcher, ‘you don't necessarily dress them in a patient costume’ (R2) for another, the patient role is ‘temporary’ (R18). Thus, a person with a neurogenic communication disorder was seen as a communication partner, in a learning process with nursing home staff, and people with hypertension ‘shared’ their data with professionals. For another researcher, the wishes and narrative were elicited from patients, despite involuntary care, when you ‘open yourself up to start a trustworthy relationship’ (R13). For several researchers, staff seemed predisposed to PCC:

There have been a few staff who've been fantastic; they had the personality for it. There, it almost felt that we were superfluous. Perhaps the reason they chose to be involved is that they're interested (R3)

A successful approach allowed for flexibility to practice in a chosen way that created the right atmosphere and space for PCC.

### Delivery of research to facilitate PCC

Several researchers described well‐trained managers who took responsibility for the PCC intervention. They transferred ideas, made things clear from the start of the project and took responsibility for monitoring professionals’ approaches and follow‐up. For the index project in particular, this process led to individual health plans, agreed in partnership between patient and professional in acute care, documented and continued into primary care.

Researchers described the way participants took an active role in the research. People who were seen as capable and resourceful partners in the research projects, facilitated the recruitment of participants, the implementation of PCC and the exchange of knowledge between professionals and persons. For example, reference groups of older people helped with training materials for active ageing or participants gave feedback on the effectiveness of keeping a graph of IBS symptoms or shared their perceptions around hypertension to develop tools. As one researcher suggested:

the narrative in this project has been there from the start in the design process, it's all about narrative, about how you feel and your situation, like, you are, they were expressing their perceptions around hypertension in the focus groups and what they felt should be included in this follow‐up instrument and why (R18)

Family members acted as a social resource for when psychosis symptoms abated or translated for relatives with communication difficulties. This participation, in turn, either facilitated the existing PCC intervention or aided future design. Researchers suggested that, despite aphasia, residents taking part in video recordings enabled professionals to watch the recordings and become better communicators. Patients’ wishes were obtained, despite involuntary care:

I think it's fantastic, just the idea that we can even for those persons who are receiving, recipients of involuntary care, that we're trying to say ‘We want to know how you want your care within this, the obvious walls that are placed around you for your safety and the safety of others, we want to try to make this care is as much in line with your wishes as possible (R15)

Researchers described an adaptive process in the delivery of PCC. For example, the translation of material and documents helped facilitate the intervention for older migrants. Moreover, although certain PCC population characteristics were described as a barrier to the intervention, other characteristics were facilitative whereby patients were acknowledged as capable, active, motivated and/or adherent. Although, as a result of these characteristics, the effects of PCC were improved, for several researchers a broader recruitment reach was desirable.

## Discussion

Authors call for a joined‐up approach to PCC, involving patients, professionals and managers, with an emphasis on patients’ priorities and goals in the context of routine care 10, 28, 29, 30, 31. Our study shows how professionals from diverse clinical backgrounds collaborate with professionals and patients to employ person‐centred research. In keeping with previous studies, we identify organisational and cultural practices in health care that restrict PCC and skilful, supported professionals who are facilitative 7, 8.

We describe how researchers are helped or hindered when developing PCC interventions in practice. Organisational systems, professional attitudes and factors associated with the delivery of their research, worked for and against PCC. Research suggests that paternalism and organisational elements in Swedish health care prevent patient participation despite legal requirements 32. However, this study details examples from practice where barriers are overcome through multidisciplinary team working, across organisations. Seeing patients and professionals as equal co‐workers combats biomedical traditions. Routinised practices and communication strategies are changed to fit and structure PCC within everyday work where patients’ not professional goals are prioritised. This level of involvement in PCC research can be both rewarding and a protracted process. Embedding PCC into existing health systems takes time.

Interviewees cited leadership and training in PCC communication as important facilitators where they practiced communicating in a different way from ‘usual’ care. For example, they employed an open questioning style, elicited the person's narrative, used effective listening skills and encouraged patient's to actively participate. For most researchers, GPCC provides the foundations and support. Trained project managers serve as reminders of the practice and philosophy of PCC. GPCC routines of narrative and partnership are necessarily adapted and adopted to fit the needs of patients with diverse conditions whereby the narrative (which may be nonverbal) or the partnership and narrative are achieved through collaboration with relatives or peers. This study contributes to the theory that PCC needs to be sensitive to the specific care context, such as the psychiatric setting, where coercion may be prevalent 33. However, documentation, as a routine for GPCC, proves particularly challenging for some projects. Most projects require more time to redevelop or change traditional ways of documenting care.

### The methods strengths and limitations

A strength of this study is the diversity of healthcare settings, bringing a broad range of experiences and methodologies connected with the development of PCC under scrutiny. Its limitations are that as the projects studied are at different stages of development and implementation, and some interviewees’ draw on past experiences and abstract or hypothetical situations. Moreover, patients were not interviewed for this research study.

In order to address these limitations research to support the extension of this work is currently underway. This includes the analysis of 18 additional interviews with professionals who are more established with the implementation of PCC and interviews with 20 patients who have experienced the GPCC model of PCC

## Conclusion

PCC has the potential to transform patient care and clinical practice. However, for the PCC approach to be successful, there needs to be a power shift and a mindset change to allow the space, time and opportunity to focus on the narrative and partnership. Explicit and consistent leadership is facilitative. However, documenting the narrative remains problematic when faced with established systems. Although this study presents a particular PCC model facilitated and implemented at GPCC, the findings provide useful guidance for developing PCC interventions across different healthcare settings. Interventions need to be adapted to fit the needs of both patients and professionals. PCC leadership and training can help address generic barriers and facilitate changes to traditional practices, systems and attitudes. Professionals, who are trained and committed to the GPCC model, can motivate others to implement PCC in other healthcare settings, therefore maintaining the continuity and quality of the implementation. This research demonstrates that developing PCC interventions and integrating these into practice takes time and professionals need on‐going support to establish and maintain PCC.

## Author contributions

All authors contributed to the study design, conception and development. Mark Elam, Axel Wolf, Oncel Naldemirci and Doris Lydahl conducted the interviews. Lucy Moore and Nicky Britten initially analysed the data, and all authors met as a group to discuss, revise and confirm the findings. Lucy Moore drafted the manuscript, and all authors were responsible for critical revision and finalising the manuscript.

## Ethical approval

The ethical code of conduct was followed and conformed to the ethical guidelines adopted by the Swedish Research Council. In accordance with Swedish ethical protocol, no ethical approval was required. However, all participants gave their informed consent and were assigned a number to ensure anonymity.

## Funding

This research was supported by the Centre for Person‐Centred Care at the University of Gothenburg (GPCC), Sweden. GPCC is funded by the Swedish Government's grant for Strategic Research Areas, Care Sciences (Application to Swedish Research Council No. 2009‐1088) and cofunded by the University of Gothenburg, Sweden; and LETS Studio – a strategic research initiative promoting interdisciplinary research within the Learning Sciences at the University of Gothenburg.
